# Hemp Seed-Derived Exosomes Protect Against Dihydrotestosterone-Induced Chicken Feather Growth Inhibition

**DOI:** 10.3390/ph18081192

**Published:** 2025-08-13

**Authors:** Hwapyung Kim, Gwangpyung Kim, Namsoo Peter Kim, Boyong Kim

**Affiliations:** 1 Department of Plant Medicals, Gyeongkuk National University, Andong 36729, Republic of Korea; kimhp031107@gmail.com; 2 Department of Biological Science, Gyeongkuk National University, Andong 36729, Republic of Korea; kimgp050321@gmail.com; 3 VIZO Investment, Washington, DC 20001, USA; nsking21@gmail.com; 4 VIZO Materials Convergence Foundation, Andong 36729, Republic of Korea; 5 EVERBIO, 131, Jukhyeon-gil, Gwanghyewon-myeon, Jincheon-gun 27809, Republic of Korea

**Keywords:** alopecia, feather formation, chicken embryo, dihydrotestosterone, callus exosomes, hemp

## Abstract

**Background/Objectives:** Androgenetic alopecia suppresses hair follicle growth. This occurs via dihydrotestosterone (DHT), which inhibits key molecular pathways such as Wnt/β-catenin and Sonic Hedgehog (SHH) signaling. Exosomes derived from plant callus cultures are promising biomaterials for targeted delivery and regenerative medicine. This study aimed to investigate the protective effects of hemp seed callus-derived exosomes (E40) against DHT-induced inhibition of feather follicle development in a chicken embryo model. **Methods:** E40 exosomes were isolated and purified from the calli of germinated hemp seeds. A DHT-induced feather loss model was established by injecting chicken embryos on embryonic day 7 (E7) with DHT (50 ng/mL), with or without co-administration of E40 (40 µg/mL). On embryonic day 12 (E12), feather length, density, and expression of molecular markers were analyzed. The methods included FISH, Western blotting, and quantitative analysis of PTCH1, AR, SHH, SMO, GLI1, Wnt, β-catenin, BMP4, and Noggin. **Results:** DHT treatment significantly reduced feather length and density. It also downregulated SHH and Wnt/β-catenin markers, upregulating BMP4 and androgen receptor expression. Co-treatment with E40 restored feather length and density to levels comparable to controls and significantly recovered the expression of SHH, SMO, GLI1, Wnt, and β-catenin. E40 also suppressed DHT-induced BMP4 upregulation by approximately 30% and reduced androgen receptor expression. **Conclusions:** These results suggest that hemp seed-derived exosomes (E40) effectively mitigate DHT-induced feather follicle inhibition by modulating critical signaling pathways and immune-related markers, supporting their potential application as a nanocarrier-based therapeutic strategy for alopecia management.

## 1. Introduction

Dihydrotestosterone (DHT) is a potent androgen hormone formed from testosterone via 5α-reductase, contributing to androgenetic alopecia by binding to androgen receptors in the feather follicle dermal papilla cells. This interaction triggers molecular signals that shorten the feather growth phase and miniaturize follicles, resulting in feather thinning and loss [1]. DHT is also associated with producing reactive oxygen species (ROS). These ROS accelerate follicle miniaturization and scalp inflammation. Oxidative stress disrupts the hair cycle. Antioxidants have been suggested as potential therapeutic agents for mitigating the effects of DHT-induced ROS, highlighting the role of oxidative stress in androgenetic alopecia pathogenesis [2]. Studies have shown that E40 increases PTCH1 expression and that DHT levels are significantly elevated in scalp regions affected by alopecia, even when systemic androgen levels remain within the normal range [3]. This localized increase is attributed to heightened 5α-reductase activity in feather follicles, particularly in individuals with a genetic predisposition to alopecia [4]. The inhibition of DHT production or its binding to androgen receptors has been proven effective in preventing the progression of feather loss. Pharmacological agents such as finasteride, a 5α-reductase inhibitor, have shown substantial efficacy in reducing scalp DHT levels and improving feather density in patients with androgenetic alopecia [5]. Additionally, studies have highlighted the role of DHT in inducing inflammatory cytokines, such as interleukin 6, which further aggravates follicular miniaturization and feather loss [6]. Recent research has also explored the modulation of DHT pathways through bioactive compounds, such as those derived from plant extracts of saw palmetto and hemp-based materials, such as E40, which show promising effects in reducing DHT-induced feather follicle damage [7]. These findings underscore the central role of DHT in the progression of alopecia and highlight the potential for targeted therapies to mitigate its effects. Consequently, ongoing studies on molecular signaling pathways, genetic modulation, and novel therapeutic agents are essential for advancing effective alopecia treatments.

E40 is a bioactive exosome derived from the calli of germinated hemp seeds. It has been recognized for its potent biological effects on cellular modulation [8]. Recent studies have demonstrated E40’s efficacy in preventing alopecia by targeting the feather follicle, dermal papillary stem cells, and immune cells [8]. E40 effectively upregulates key feather growth-promoting genes such as *Wnt*, *β-catenin*, and *TCF*, while downregulating alopecia-inducing genes like *STAT1*, *IL-15R*, and *NKG2DL* [8]. This dual-action mechanism mitigates DHT-induced stress, fosters feather follicle regeneration, and restores immune homeostasis [8]. E40 exhibited superior efficacy in promoting cellular differentiation and modulating immune responses compared to other hemp-derived materials. Its enhanced potency compared to conventional treatments highlights its potential as a therapeutic agent for androgenic alopecia. E40’s influence on gene expression, particularly in the Wnt/β-catenin pathway, underscores its role in feather follicle cycling and regeneration [8]. Additionally, E40 suppresses pro-inflammatory cytokines such as interferon γ, further reducing immune-related feather loss. The promising bioactive properties of E40 make it a valuable candidate for pharmaceutical development for the treatment of alopecia [8]. Recent studies have demonstrated that exosomes derived from mesenchymal stem cells and plant sources can promote hair follicle development and skin regeneration by modulating key signaling pathways such as Wnt/β-catenin and Hedgehog signaling [9,10]. Furthermore, plant-derived exosome-like nanovesicles were shown to regulate immune responses through miRNA cargo, supporting our current rationale [11,12]. Future studies exploring its in vivo effects are crucial for unlocking its full therapeutic potential.

Alopecia is a multifactorial condition influenced by various signaling pathways and molecular markers. Among them, patched 1, androgen receptor, sonic hedgehog (SHH), smoothened (SMO), Wnt, β-catenin, Noggin, and bone morphogenetic protein 4 (BMP4) play crucial roles in feather follicle regulation and feather growth cycles. PTCH1 is a key receptor in the SHH signaling pathway, vital for feather follicle morphogenesis and regeneration. PTCH1 mutations or dysfunction can impair SHH signaling, resulting in feather follicle miniaturization, feather cycle disruption, and contributing to alopecia [13]. Through its interaction with SMO, the SHH pathway promotes the proliferation of feather follicle stem cells and initiates the anagen phase, which is essential for feather growth [14]. Androgen receptors are closely associated with androgenic alopecia. DHT binds to AR and activates gene expression, promoting feather follicle miniaturization and shortening the anagen phase [1]. Elevated AR expression in scalp feather follicles is strongly associated with an increased susceptibility to DHT-induced feather loss [6]. The Wnt/β-catenin signaling pathway is pivotal in promoting feather follicle regeneration and activating feather follicle stem cells. Activation of Wnt signaling leads to β-catenin accumulation in the nucleus, triggering the expression of genes responsible for feather growth. Dysregulation or suppression of this pathway is linked to feather loss and its progression [15]. Noggin is a BMP antagonist that protects feather growth by inhibiting BMP4. BMP4 inhibits the activation of feather follicle stem cells and is involved in the maintenance of feather follicles during the telogen (resting) phase. Elevated BMP4 expression has been linked to prolonged telogen phase and feather loss [16]. Noggin counteracts BMP4 signaling, promotes feather follicle cycling, and delays follicular miniaturization. The interplay between these markers demonstrates complex regulatory mechanisms governing feather follicle development, cycling, and regeneration. Thus, therapeutic strategies targeting these pathways, particularly enhancing Wnt/β-catenin and SHH signaling while inhibiting AR and BMP4, offer promising directions for treating alopecia.

The chick embryo model is widely used in developmental biology owing to its accessibility, cost-effectiveness, and physiological similarities to human skin development. During feather formation in chick embryos, the E7 (embryonic day 7) and E12 (embryonic day 12) stages are particularly crucial. At E7, feather placodes, which are specialized epithelial structures that give rise to feathers, begin to form through interactions between the epidermis and dermis. This stage is vital for initiating the feather bud pattern, which is regulated by molecular pathways such as Wnt, BMP, and SHH signaling [17]. By E12, these placodes differentiate into defined feather buds with organized growth zones and early morphological features resembling mature feathers. This stage is critical for evaluating feather follicle development, cellular differentiation, and dermal–epithelial interactions [18]. In preclinical studies on feather loss treatments, chick embryo models provide a valuable alternative before conducting human trials. The structural and molecular similarities between chick and human feather follicles make this model highly suitable for testing bioactive materials, growth factors, and signaling pathway modulators. Moreover, chick embryos allow the rapid evaluation of cellular responses to treatments, enhancing our understanding of follicle regeneration processes. Using chick embryos in preclinical trials is essential for identifying effective treatments while minimizing risks and ethical concerns in early-phase human testing [19]. Using E12 chick embryos in experiments offers a significant ethical advantage because they are considered non-sentient organisms before hatching [20]. According to international guidelines, such as those set by the Institutional Animal Care and Use Committee (IACUC), chick embryos up to E14 are generally exempt from IACUC reviews because they are not yet capable of experiencing pain or distress. This exemption allows researchers to efficiently conduct developmental and pharmacological studies while adhering to ethical research standards [21,22].

This study validated the preventive effects of E40 exosomes derived from the callus tissues of germinated hemp on feather loss through in vitro experiments [8]. Based on these findings, we aimed to assess the clinical applicability of E40 using alternative preclinical models, such as in ovo models. By ensuring consistency across preclinical results, this study provides scientific evidence supporting the efficacy of E40, thereby contributing to the industrialization of functional materials for alopecia prevention and the development of alternative non-animal testing platforms.

## 2. Results

### 2.1. Alopecia Modeling Using Chick Embryos and E40 Injection Dosage Determination

To establish a reliable in ovo model of feather growth inhibition, we first examined the dose-dependent effects of DHT on feather length and TGF-β1 expression in chick embryos.

**Figure 1:**
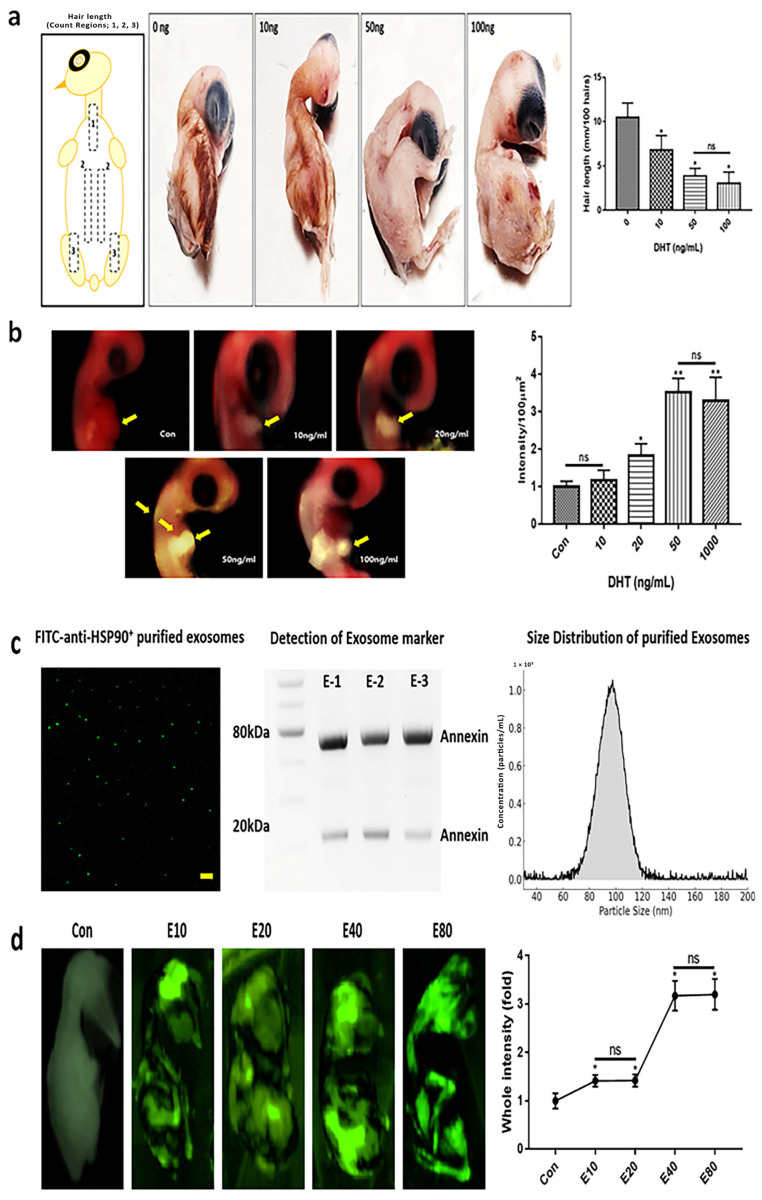
Modeling of feather deficiency in chicken embryos using DHT. (**a**) Changes in feather length of chicken embryos (E12) according to the concentration of dihydrotestosterone (DHT). (**b**) Expression levels of TGF-β1 in dermal papilla cells of chicken embryos collected at E8 (24 h after DHT injection at E7), reflecting early molecular responses to androgen exposure. (**c**) Confirmation of purified exosomes through fluorescence staining using FITC-Anti-HSP90 and Anti-Annexin antibodies, followed by Western blot analysis. E-1 to E-3 refer to three different samples of exosomes that were purified using the same method. Representative nanoparticle tracking analysis (NTA) demonstrating the size distribution profile of purified exosomes. Data indicate a primary peak with a mean particle diameter of approximately 95 nm. (**d**) Determination of the optimal exosome injection concentration by quantitatively analyzing fluorescence signal intensity and area using the iBright FL1000 system and iBright Analysis Software (version 4.0.1). The condition with an evenly distributed signal without over-saturation was selected as optimal. Con: control; ns: not significant; yellow arrows: positive regions; (* *p* < 0.05, ** *p* < 0.01) (scale bar = 10 μm).

Feather formation was examined after injecting chicken embryos with varying concentrations of DHT on day 7 (E7) and culturing until day 12 (E12). E7 embryos were selected for substance injection, as feather placode formation begins at this stage and the yolk sac remains accessible for effective delivery. To evaluate early transcriptional responses, especially TGF-β1 expression, embryos were collected at E8, allowing sufficient time (24 h post-injection) for molecular changes. Significant feather deficiency was observed at DHT concentrations of 50 and 100 ng/mL (Figure 1a). Furthermore, the expression of transforming growth factor-β1 corresponded with the observed DHT concentration-dependent effects (Figure 1b). Taken together, these findings suggest that the optimal concentration of DHT for modeling feather deficiency in chicken embryos is 50 ng/mL (Figure 1). To confirm the purification of plant-derived exosomes, an analysis was performed using antibodies against HSP90 and Annexin proteins, which are commonly expressed in both animal and plant exosomes. Notably, Annexin-like proteins derived from E40 exosomes exhibited bands near 25 and 70 kDa, distinct from the typical ~36 kDa Annexin A1 observed in mammalian systems. These findings are consistent with previous reports indicating that plant-derived extracellular vesicles contain annexin isoforms or modified forms with variable molecular weights, particularly within the 20–80 kDa range (Figure 1c) [23,24]. Nanoparticle tracking analysis (NTA) revealed a narrow size distribution for the purified exosomes, with the predominant particle population exhibiting an average size of 95 ± 6 nm. The concentration of exosomes at the peak was approximately 1.0 × 10^8^ particles/mL, indicating successful purification with minimal contamination from larger particles or aggregates (Figure 1c). FITC-Anti-HSP90-stained exosomes were injected into chicken embryos to determine appropriate exosome injection concentrations. The results showed similar fluorescence intensities for E40 and E80, suggesting that E40 was the optimal injection concentration (Figure 1d). The fluorescent signal was quantitatively assessed using the iBright FL1000 imaging system (Invitrogen, Thermo Fisher Scientific, Waltham, MA, USA). Signal intensity and area were measured using iBright Analysis Software (version 4.0.1), and the optimal exosome concentration was defined as the condition exhibiting uniform fluorescence distribution without saturation or aggregation.

Based on the optimized DHT concentration and validated E40 injection dose, we next investigated the protective effects of E40 on feather growth parameters, such as length and density, in the DHT-induced alopecia model.

### 2.2. The Protective Effect of E40 Against DHT and Its Impact on Feather Length and Density

After determining the optimal DHT concentration and confirming the feasibility of the exosome injection, we evaluated the protective effects of E40 on feather length and density in a DHT-induced model.

**Figure 2:**
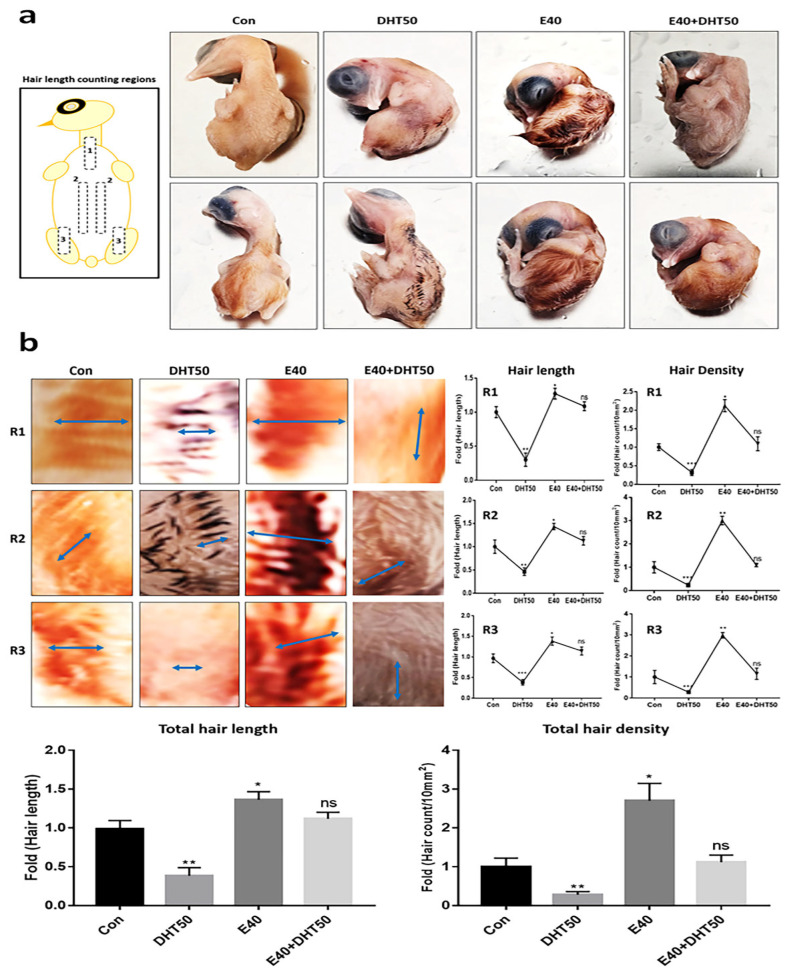
Effects of hemp-derived callus exosomes on DHT-induced inhibition of feather formation. (**a**) Protective effect of E40 (hemp seed callus-derived exosomes, 40 µg/mL) against feather formation inhibition under DHT50 (50 ng/mL) conditions. (**b**) The results of evaluating feather length (arrows; average measured lengths) and density in the three regions of E12 exposed to the various conditions; Con: control; DHT50, E40, E40 + DHT50: mixture; ns: not significant (* *p* < 0.05, ** *p* < 0.01, *** *p* < 0.001).

Compared to normal embryos, feather length in embryos treated with DHT50 was reduced by approximately 44% across the three regions (Figure 2a), and a noticeable decrease in feather density was visually observed (Figure 2b). Remarkably, in chicken embryos injected with E40, feather length and density increased approximately 1.32 and 2.81 times, respectively (Figure 2b). Furthermore, despite the DHT50-induced stress, embryos treated with E40 exhibited feather lengths comparable to those of normal embryos (Figure 2a,b).

To explore the molecular basis of E40’s protective effects, we analyzed key receptors and signaling molecules involved in feather follicle morphogenesis.

### 2.3. Evaluation of the Molecular Effects of E40 on Feather Length and Density

**Figure 3:**
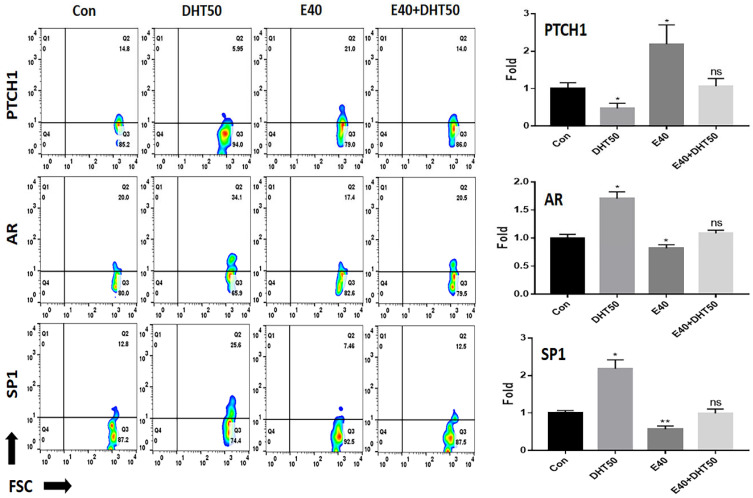
Effect of E40 on the expression of signal receptor markers in feather formation. Fluorescence in situ hybridization (FISH) analysis using fluorescently labeled anti-mRNA probes for each marker (PTCH1, AR, and SP1). Con: control; DHT50: dihydrotestosterone 50 ng/mL. E40: hemp seed callus-derived exosomes, 40 µg/mL; E40 + DHT50: mixture; ns: not significant (* *p* < 0.05, ** *p* < 0.01).

**Figure 4:**
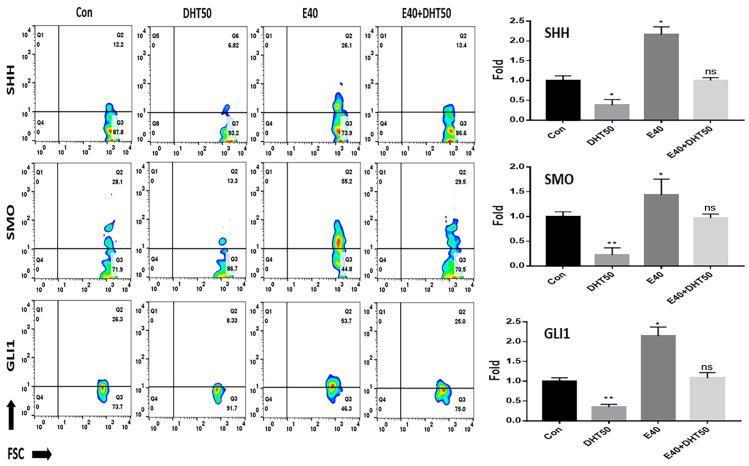
Effect of E40 on the expression of upstream markers in feather formation. Fluorescence in situ hybridization (FISH) analysis using fluorescently labeled anti-mRNA probes for each marker (SHH, SMO, and GLI1). Con: control; DHT50: dihydrotestosterone 50 ng/mL. E40: hemp seed callus-derived exosomes, 40 µg/mL; E40 + DHT50: mixture; ns: not significant (* *p* < 0.05, ** *p* < 0.01).

To elucidate the molecular mechanisms underlying E40’s effects, we next analyzed gene expression changes in key signaling pathways involved in feather morphogenesis, including SHH, Wnt/β-catenin, and BMP4. Evaluation of PTCH1 expression, a crucial receptor that interacts with the SHH ligand to promote feather formation in chicken embryos, revealed that E40 increased PTCH1 expression by approximately 2.2 times compared to the control. Despite the presence of DHT, PTCH1 expression was similar to that in the control group (Figure 3). *AR* mRNA levels increased in response to DHT, which binds to the AR, and the expression of SP1, an AR transcription factor, showed a pattern of change similar to that of AR (Figure 3). Analysis of the SHH signaling pathway, which is crucial for feather formation in chicken embryos, revealed that the mRNA levels of the early downstream markers SHH, SMO, and zinc finger protein GLI1 (GLI1) were significantly suppressed by DHT50 treatment, with an average reduction of approximately 64% compared to normal embryo cells. E40 treatment increased these mRNA levels by approximately 1.85 times on average, effectively restoring their expression levels to those observed in normal embryos, despite the presence of DHT50 (Figure 4).

**Figure 5:**
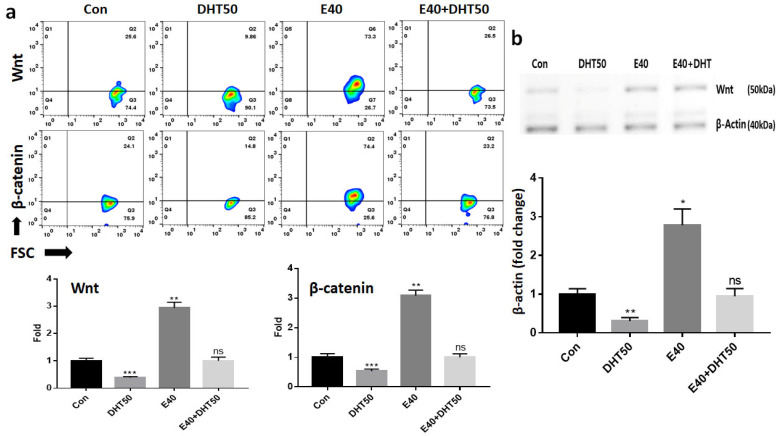
Effect of E40 on the expression of middle stream markers in feather formation. (**a**) Fluorescence in situ hybridization (FISH) analysis using fluorescently labeled anti-mRNA probes for each marker (Wnt and β-catenin). (**b**) Western blot analysis of Wnt protein expression levels. Band intensities were quantified using iBright FL1000 (Invitrogen, Thermo Fisher Scientific) and iBright Analysis Software (version 4.0.1). All values were normalized to β-actin expression levels, and the data represent relative fold-change from three independent experiments. Con: control; DHT50: dihydrotestosterone, 50 ng/mL; E40: hemp seed callus-derived exosomes, 40 µg/mL; E40 + DHT50: mixture; ns: not significant (* *p* < 0.05, ** *p* < 0.01, *** *p* < 0.001).

Having confirmed the rescue of SHH signaling components, we investigated whether E40 also influences midstream regulators such as Wnt and β-catenin, which are essential for feather bud activation. Evaluation of the mRNA levels of midstream markers revealed that DHT50 treatment resulted in an average reduction of approximately 67% compared with the control group. However, in embryos treated with E40, the mRNA levels increased by approximately 3.1-fold relative to the control group and were maintained at levels comparable to those of the control group, even in the presence of DHT50 (Figure 5a). After confirming the recovery of SHH pathway components by E40, we assessed their influence on midstream markers involved in feather follicle development, particularly Wnt and β-catenin, which are central to follicular activation and anagen phase maintenance. Given that Wnt and β-catenin are key markers of feather formation, Western blotting was performed to assess their protein expression levels (Figure 5b). The results demonstrated an approximately 2.8-times increase in protein expression compared to the control group. This suggests that E40 potentially rescues feather growth through anti-androgenic mechanisms or by delivering pro-growth signals via exosomal cargo, including specific miRNAs and proteins. These results closely mirrored the mRNA analysis findings, cross-validating the observed gene expression patterns (Figure 5b).

**Figure 6:**
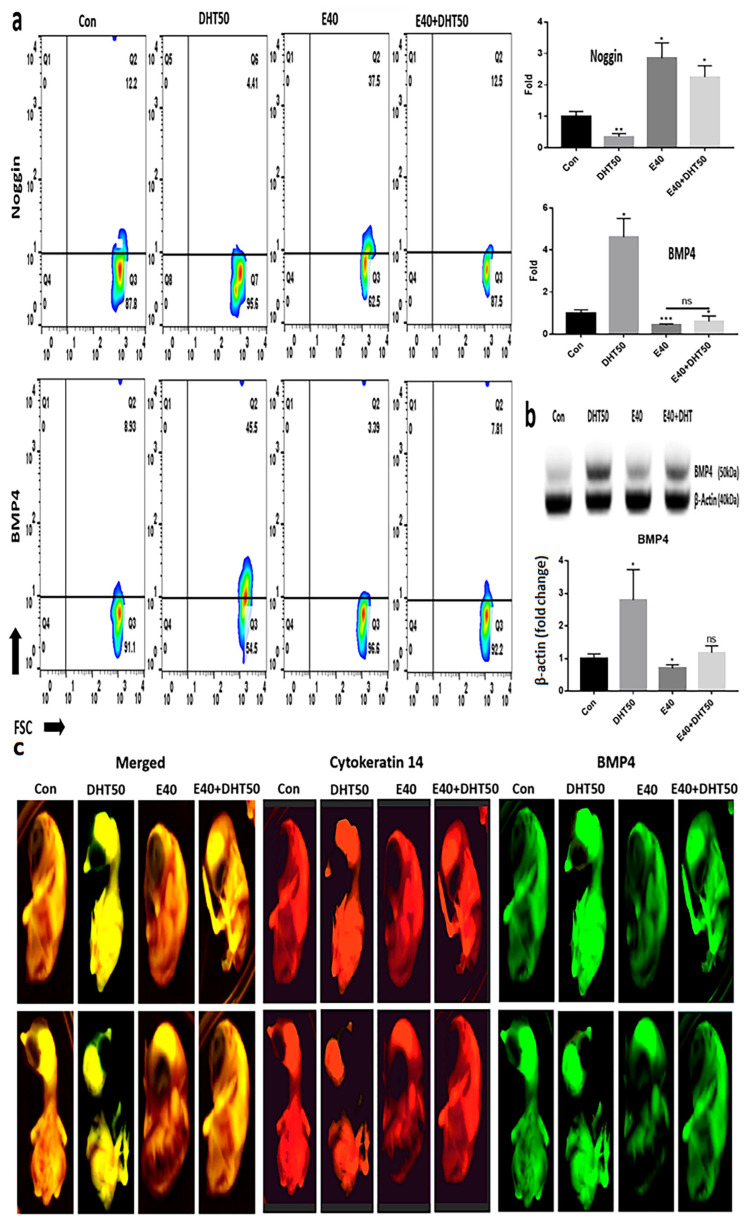
Effect of E40 on the expression of downstream markers in feather formation. (**a**) Fluorescence in situ hybridization (FISH) analysis using fluorescently labeled anti-mRNA probes for each marker (Noggin and BMP4). (**b**) The results of expression levels for BMP4 using Western blotting. The analysis was normalized to beta-actin expression. (**c**) FISH results using the cytokeratin 14 gene probe (red), bone morphogenetic protein 4 (BMP4) gene probe (green) and two positives (yellow); Con: control; DHT50: dihydrotestosterone 50 ng/mL. E40: hemp seed callus-derived exosomes, 40 µg/mL; E40 + DHT50: mixture; ns: not significant (* *p* < 0.05, ** *p* < 0.01, *** *p* < 0.001).

Because inhibitory signals also play a critical role in feather cycling, we assessed the modulation of BMP4 and its antagonist, Noggin, to determine E40’s broader regulatory effects on follicle regeneration. In addition to activation pathways, inhibitory signals such as BMP4 are crucial regulators of follicle cycling. Therefore, we investigated whether E40 modulates BMP4 expression and its antagonist Noggin, which counteracts BMP-mediated suppression of feather development. Evaluating BMP4, a key protein that plays a crucial and direct role in feather development in chicken embryos, revealed significant changes in response to DHT50 treatment. Compared to the mRNA levels of Noggin and BMP4 in normal embryonic cells, DHT50 treatment resulted in an approximately 80% reduction in mRNA expression (Figure 6a). Cross-validation of BMP4 protein expression confirmed a 2.8-times increase in BMP4 levels following DHT50 treatment (Figure 6b). In the presence of E40, the BMP4 protein expression was reduced by approximately 30% (Figure 6b). The staining results of embryos exposed to the four different conditions using the BMP4 probe showed a pattern similar to that observed in Figure 6a,b (Figure 6c).

## 3. Discussion

The results of this study reveal the potential of hemp seed callus-derived exosomes (E40) as novel therapeutic agents for combating DHT-induced alopecia. Hemp exosomes have previously been reported to exhibit regenerative properties, particularly in modulating cellular proliferation, differentiation, and immune regulation [7]. The findings of the present study are consistent with those of previous studies. E40 enhances hair follicle stem cell differentiation. It also suppresses the key pathways that promote alopecia. The protective mechanism of E40 against DHT-induced stress was particularly noteworthy. DHT is known to trigger feather follicle miniaturization by binding to androgen receptors, suppressing growth-stimulating pathways such as Wnt/β-catenin and SHH signaling [1,8]. E40 successfully mitigated these effects by restoring PTCH1 expression, a vital receptor in the SHH pathway that promotes feather follicle morphogenesis [13]. Restoration of PTCH1 by E40 suggests its ability to rescue the SHH signaling cascade, which is typically suppressed under DHT-stress conditions [14]. In addition to PTCH1 restoration, E40 effectively enhanced the expression of downstream markers such as SMO and GLI1, which are crucial for activating SHH signaling [15]. This restoration aligns with reports that the SHH pathway activation plays a fundamental role in feather regeneration and cycling [17]. The ability of E40 to upregulate the Wnt/β-catenin pathway, a critical regulator of feather follicle development, further reinforces its potential as a therapeutic candidate for alopecia. Previous studies have emphasized that the Wnt/β-catenin pathway is essential for feather follicle growth and maintaining the anagen phase, making it a promising target for feather loss treatments [15]. E40 suppressed BMP4 expression. This indicates an additional protective mechanism. As BMP4 prolongs the resting (telogen) phase and inhibits stem cell activation, its reduction by E40 may help to reinitiate the hair growth cycle. BMP4 inhibits feather follicle stem cell activation and prolongs the telogen phase, contributing to feather thinning and loss [16]. By reducing BMP4 expression by approximately 30%, E40 demonstrated the ability to shift feather follicle cycling back to the active growth phase, thereby enhancing overall feather density and length. This effect may be mediated by two mechanisms: (1) inhibition of androgen receptor signaling; (2) activation of pro-growth signaling via the exosomal cargo of E40, including miRNAs and proteins. These components likely contribute to the restoration of the SHH and Wnt/β-catenin pathways and the suppression of BMP4 and AR expression, as observed in our molecular analyses [25].

Importantly, E40 displayed potent immune-modulating properties in CD8+ T cells by downregulating inflammatory markers such as NKG2DL, IL2-Rβ, and JAK1. This immune regulatory function aligns with recent findings that immune dysregulation exacerbates feather loss in patients with alopecia [8]. By restoring the immune balance, E40 offers an additional pathway for protecting feather follicle health under DHT-induced stress. Considering the molecular outcomes observed in this study, E40 has substantial potential for clinical applications. Its superior efficacy compared with that of germinated hemp seed extract demonstrates its potential as a therapeutic agent for prevention and treatment [8]. The demonstrated ability of E40 to target multiple signaling pathways, including SHH, Wnt/β-catenin, and BMP4 suppression, provides compelling evidence for its multifaceted mechanism in promoting feather regeneration. Future studies should focus on expanding E40 research to include in vivo mammalian models to confirm its therapeutic efficacy in chicken embryo models. In addition, investigating the optimal dosage, treatment duration, and long-term safety profile is critical for E40’s successful development as a pharmaceutical or cosmeceutical product. Furthermore, exploring E40’s potential synergy with existing alopecia treatments such as minoxidil and finasteride may enhance its clinical utility. In conclusion, this study highlighted the novel application of E40 as a potential therapeutic candidate for DHT-induced alopecia. E40’s ability to modulate molecular pathways, regulate immune responses, and promote feather follicle regeneration underscores its potential for clinical and commercial applications. Given the increasing global demand for innovative feather loss treatments, E40 is a promising bioactive material with high potential to revolutionize alopecia management strategies.

Previous studies have demonstrated that exosomes derived from mesenchymal stem cells or dermal papilla cells contribute to hair follicle regeneration by delivering bioactive molecules, such as miRNAs, Wnt-related proteins, and growth factors. For instance, recent studies showed that human follicle dermal papilla cell-derived exosomes promote hair growth by activating the Wnt/β-catenin pathway [26,27]. Similarly, exosomes from adipose-derived stem cells have been reported to accelerate anagen entry and stimulate dermal papilla cell proliferation via miR-22 and VEGF signaling [28,29]. E40’s remarkable efficacy may extend beyond signaling pathway modulation to mechanisms such as exosome-mediated delivery systems and cell-to-cell interactions. Future studies should explore these mechanisms to develop optimal therapeutic approaches. Furthermore, E40’s activation of Wnt/β-catenin and SHH pathways is closely related to wound healing and tissue regeneration mechanisms, suggesting broader clinical applications beyond feather restoration. Additionally, E40’s gene modulation capabilities could be integrated with gene therapy techniques to provide a more stable and long-term treatment strategy for alopecia. Finally, the development of personalized treatment approaches based on E40’s diverse molecular mechanisms and immune-regulating properties could help to tailor therapies to patients’ genetic profiles and immune conditions, thereby improving treatment efficacy. Despite promising findings in the chicken embryo model, it is important to acknowledge its limitations in fully mimicking mammalian feather follicle biology [30]. The structural, immunological, and hormonal differences between avian and mammalian feather follicles warrant further in vivo validation [30]. Accordingly, future research using well-established mammalian models such as C57BL/6 mice, followed by clinical trials, is essential to verify the safety, efficacy, and applicability of E40 in human feather loss therapy [31]. Although structural observations via paraffin sectioning were not conducted in this study, future investigations will include histological examination of feather follicles to support the phenotypic findings with anatomical evidence. Additionally, future research should focus on optimizing E40 dosage and treatment duration and identifying patient-specific factors to maximize efficacy. Because hemp-derived exosomes have emerged as bioactive agents, E40’s robust molecular effects position it as a strong candidate for commercial development in feather regrowth therapies and regenerative medicine. With further research on its mechanistic pathways and safety profile, E40 may become a powerful tool for combating alopecia and enhancing dermatological treatment.

## 4. Materials and Methods

### 4.1. Callus Induction and Purification of Exosomes from Germinated Hemp Seeds

After immersing 5 g of sterilized hemp seeds (Cheongsam) (88 company, Andong, Republic of Korea) in 1% H_2_O_2_ (hydrogen peroxide) for 24 h, the hydrated seeds were germinated for three days at 24 °C in Murashige and Skoog (KisanBio, Seoul, Republic of Korea) germination media containing gibberellic acid (KisanBio), 1-naphthaleneacetic acid (KisanBio), and sucrose (KisanBio). Meristematic tissues were isolated from the germinated hemp seeds and cultured in Murashige and Skoog callus-inducing medium (Kisan Bio) containing 6-benzylaminopurine (Kisan Bio), indoleacetic acid, and sucrose. After culturing the calli for five weeks in callus transfer medium, exosomes were isolated and purified from the calli using an ExoEasy Maxi Kit (QIAGEN, Hilden, Germany). Purified exosomes obtained from cultured calli were confirmed by Western blotting using an Anti-Annexin V/ANXA5 antibody (ab108194; Abcam, Cambridge, UK). The proteins extracted from the purified exosomes were subjected to 12–15% sodium dodecyl sulfate-polyacrylamide gel electrophoresis, followed by immunoblotting with an anti-Annexin V/ANXA5 antibody at a dilution ratio of 1:500. Western blotting images were captured using an iBright FL1000 imaging system (Invitrogen, Carlsbad, CA, USA). Protein band intensities were analyzed using iBright Analysis Software (version 4.0.1), and target protein levels were normalized to β-actin expression as a loading control. Exosome particle size distribution was analyzed using nanoparticle tracking analysis (NTA) with a NanoSight NS300 instrument (Malvern Panalytical, Malvern, UK). Exosome samples were diluted in filtered phosphate-buffered saline (PBS) to achieve optimal particle concentration for analysis (approximately 10^8^ particles/mL). Measurements were conducted at room temperature, and particle movement was recorded and analyzed using NanoSight NTA 3.4 software (Malvern Panalytical, Malvern, UK). Each measurement consisted of three independent runs, and results were averaged to obtain the final size distribution profile. Particle size and concentration data were presented as the mean particle diameter ± standard deviation, calculated from the Gaussian fitting of the size distribution peak.

### 4.2. Confirmation of Purified Exosomes and Determination of the Injection Concentration in Chicken Embryos

Exosomes were purified from callus tissues derived from the meristematic tissues of germinated hemp seeds, using an Exosome Purification Kit (exoEasy Maxi Kit, QIAGEN, Cat. no. 76064; Hilden, Germany). Two methods were used to confirm the presence of the purified exosomes. First, the purified exosomes were stained with anti-heat shock protein 90 (HSP90) antibody (HSP90 MA5-45103, FITC, Thermo Fisher Scientific, Waltham, MA, USA) at a dilution ratio of 1:1000 for 1 h at 37 °C, followed by observation under a fluorescence microscope (Eclipse Ts-2; Nikon, Shinagawa, Japan). To assess the distribution and intensity of injected exosomes, embryos were imaged using an iBright FL1000 fluorescence imaging system (Invitrogen, Thermo Fisher Scientific, Waltham, MA, USA). Quantitative analysis of fluorescence signal intensity and area was performed using iBright Analysis Software (version 4.0.1). The optimal exosome injection concentration was determined based on uniform signal distribution without oversaturation or aggregation. To determine the injection concentrations of the chicken embryos, purified exosomes were first stained with FITC-Anti-HSP90 antibody at a dilution ratio 1:2000 for 1 h at 37 °C. Stained exosomes were injected into the yolk sac of E7 chicken embryos. Subsequently, at the E12 stage, the presence and distribution of exosomes in chicken embryos were tracked using an FL1000 system (Thermo Fisher Scientific).

### 4.3. Incubation of Chick Embryos

Fertilized *Gallus gallus domesticus*, Hy-Line Brown eggs (Eco Farm, Jincheon, Republic of Korea) were purchased and incubated at 37 °C with 70–90% humidity. Experiments were conducted on E7, and the injection of substances (Con; control, DHT50; dihydrotestosterone 50 ng/mL E40; hemp seed callus-derived exosomes, 40 µg/mL, E40 + DHT50; mixture) was performed via yolk injection, with an injection volume of 200 µL. For in ovo administration, 200 µL of hemp seed-derived exosomes (E40) [8] was injected into the yolk sac of fertilized chicken embryos on embryonic day 8 (E8). The injection solution contained E40 at a 40 µg/mL concentration, corresponding to approximately 1 × 10^9^ exosomal particles, as determined using a commercial exosome standard (System Biosciences, Palo Alto, CA, USA) [32]. This dose was selected based on our previous study that demonstrated the bioactivity of germinated hemp seed-derived exosomes on stressed hair stem cells and immune cells [8]. Fluorescence in situ hybridization (FISH) was conducted using anti-mRNA probes (Table 1). The treatment concentrations were determined based on the effective dose (EC50). According to international animal protection regulations, including the EU Directive 2010/63/EU, USDA, and NIH standards, chick embryos before E12 can typically be used for experiments without requiring animal experimentation approval.

### 4.4. Whole-Mount Immunohistochemistry FISH

Embryos fixed in 4% paraformaldehyde for 24 h were dehydrated and cleared using a graded series of 70–100% ethanol (Sigma-Aldrich, St. Louis, MO, USA) and 100% xylene (Sigma-Aldrich). The cells were incubated in 0.4% Tween 20 (Sigma-Aldrich) for 24 h. Fluorescent probes (10 µL each) (Bioneers, Daejeon, Korea) (Table 1) were added, and staining was performed for five days. The fluorescence intensity and area of the stained embryos were analyzed using an FL1000 ChemiDoc (Thermo Fisher Scientific), and statistical analysis was conducted using Prism (GraphPad, Boston, MA, USA).

### 4.5. Measurement of Feather Length and Density in Chick Embryos

E7 chick embryos were injected under four conditions (control, DHT50, E40, and E40 + DHT50) and extracted at E12. The length and density of feathers in the three regions (Figure 1 and Figure 2) of the paraformaldehyde-fixed chick embryos (E12 and EE38) were measured (C-CUBE FEATHER; PIXIENCE, Toulouse, France).

### 4.6. Tissue and Single-Cell Dissociation Analysis

A seven-day-old chicken embryo (E7) exposed to various conditions (control, DHT50, E40, and E40 + DHT50) was aseptically dissected and washed with phosphate-buffered saline to remove blood and residual tissues at E12. Ventral skin and feather follicle tissues were excised. Isolated tissues were incubated in a mixture of collagenase IV (2 mg/mL) (Sigma-Aldrich) and dispase (1 mg/mL) (Sigma-Aldrich) at 37 °C for 30–40 min for enzymatic digestion. After blocking with 10% fetal bovine serum, the samples were centrifuged at 112× *g* for 5 min, and the pellets were treated with 0.05% trypsin–EDTA (Sigma-Aldrich) for 5–10 min to obtain a single-cell suspension. The cell suspensions were filtered using a 70 μm cell strainer, and the filtered samples were centrifuged again at 112× *g* for 5 min to obtain single cells. After exposure to a blocking buffer containing 2% bovine serum albumin (Sigma-Aldrich) for 10 min, the blocked cells were treated with 0.02% Tween 20 for 30 min. The treated cells were exposed to fluorescently labeled probes (Table 1), and the stained cells were evaluated for marker levels using a flow cytometer (BD Biosciences, San Jose, CA, USA) and FlowJo (BD Biosciences).

### 4.7. Statistics

All quantitative data were analyzed using one-way analysis of variance (ANOVA) followed by Scheffé’s post hoc test for multiple comparisons. Statistical analyses were performed using Prism 7 software (GraphPad Software, Boston, MA, USA). Results are presented as the mean ± standard deviation (SD) from at least three independent experiments. Statistical significance was defined as *p* < 0.05. In all figures, significance levels are indicated by asterisks: *p* < 0.05 (*), *p <* 0.01 (**), and *p <* 0.001 (***). Each group included eight embrnyos (*n* = 8) per condition, and all in ovo experiments were independently repeated at least three times to ensure reproducibility.

## 5. Conclusions

This study highlights the potential of hemp-derived exosomes (E40) as promising therapeutic candidates for the treatment of DHT-induced alopecia. E40 effectively restored key pathways such as PTCH1, Wnt/β-catenin, and SHH, which regulate feather follicle growth. Additionally, E40’s suppression of BMP4 and inflammatory markers such as NKG2DL and JAK1 underscores its protective role in both follicular health and the modulation of immune responses. E40 demonstrated superior efficacy over germinated hemp seed extract, reinforcing its clinical potential for alopecia treatment. Despite these promising results, the limitations of this study include its reliance on chicken embryo models, necessitating further mammalian studies to confirm efficacy and safety. Investigations on the optimal dosage, treatment duration, and potential side effects are also required. Exploring E40’s possible synergy among minoxidil and finasteride may further enhance its clinical value. In conclusion, E40’s multifaceted mechanisms, combining the molecular regulation and modulation of immune responses, make it a novel therapeutic candidate. With continued research into its mechanisms and clinical applications, E40 holds strong potential to revolutionize alopecia treatments and regenerative medicine.

## 6. Patents

**Patent**: Korean Patent No. 10-2783000.

**Title**: Bioactive material derived from hemp for the prevention and treatment of feather loss.

**Country:** The Republic of Korea.

**Status**: Registered.

This research was based in part on a patented technology titled “*Bioactive Material Derived from Hemp for the Prevention and Treatment of Feather Loss*” (Korean Patent No. 10-2783000), which has been officially registered in the Republic of Korea.

## Figures and Tables

**Table 1:** Sequences of anti-mRNA probes for FISH.

Gene Name	Sequence (5′->3′)
*SHH* (Sonic Hedgehog)	Probe1: FITC-AGAGACCGCGCAGTTCCGACGAGG Probe2: FITC-TCTGCTGGAGAGGCGTGTTAGCAG
*SMO* (Smoothened)	Probe1: PE-CCTGAGGCTGCCATGTTGGTAGAG Probe2: PE-AGTGGCTTCCAGCCTGATGAGCAA
*CTNNB1* *(Beta-catenin)*	Probe1: Cy3-ATGGAGCCGGACAGAAAAGCGGCA Probe2: Cy3-TGCAGGAGTCCAGCAGATGGCCAC
*BMP4* (Bone Morphogenetic Protein 4)	Probe1: FITC-GGAGGATGGCCAGGAGTATGCCAT Probe2: FITC-TTGACCCAGGAGGCGGACTTGGAC
*Noggin*	Probe1: PE-TGCTGGACGATGAGGCTTACCTGA Probe2: PE-AGGAGGAGTGGACAGAGTCCAGAG
*Wnt3a*, *Wnt7a* (Wingless-type MMTV integration site family member 3A, 7A)	Probe1: Cy3-GGCTGAGTGGTGGAGGATGCTCTA Probe2: Cy3-AGATGCTGAGGGAGTCCGAGGACT
*GLI1* (Glioma-associated oncogene homolog 1)	Probe1: FITC-AGAGTGCTGAGGACGAGTCTGAGA Probe2: FITC-TCTGAGGAGGTGCTGAGGTCGAGA
*Cytokeratin 14*	Probe1: Cy3-ATG GCTCCGCAATGTGCTTCTGAA G Probe2: Cy3-TGCCAGGAACTGCTCATCTGATGC
*Androgen Receptor*	Probe1: FITC-ATGCTGCTCAGTGCCTACCAAGATGACACCTGAG Probe2: FITC-GACCTGGTGCTAGATGCTGAACAGGCTCAGCTGC
*PTCH1* *(Patched 1)*	Probe1: FITC-CTGACAGCTGGTAGACCTGAGCAGAGAGACCTGA Probe2: FITC-AGTCTGGCATAGTGCCTGACAGCTGATGAGCTGA

## Data Availability

The original contributions presented in this study are included in the article. Further inquiries can be directed to the corresponding author.

## Abbreviations

The following abbreviations are used in this manuscript:

AR	androgen receptor
BMP4	bone morphogenetic protein 4
DHT	dihydrotestosterone
FISH	fluorescence in situ hybridization
GLI1	zinc finger protein GLI1
HSP90	heat shock protein 90
PTCH1	patched 1
SHH	sonic hedgehog
SMO	smoothened
